# Surveillance for invasive candidiasis in China (CHIF-NET 2018-2021): rising antifungal resistance observed in a nationwide longitudinal study

**DOI:** 10.1128/aac.00035-26

**Published:** 2026-04-27

**Authors:** Qiao-Lian Yi, Kai-Wen Xu, Peng-Hao Guo, Li Li, Ling Ma, Ya Liu, Ning Li, Xiao-Chun Huang, Hong He, Wen-Ying Xia, Jing Guan, Jin Li, Ge Zhang, Wei Kang, Jing-Jia Zhang, Tong Wang, Hao-Tian Gao, Dan Guo, Ying-Chun Xu, Meng Xiao, On behalf of the China Hospital Invasive Fungal Surveillance Net (CHIF-NET) Study Group

**Affiliations:** 1 Department of Laboratory Medicine, State Key Laboratory of Complex Severe and Rare Diseases, Peking Union Medical College Hospital, Chinese Academy of Medical Sciences and Peking Union Medical College 670119, Beijing, China; 2 Department of Clinical Laboratory, The First Affiliated Hospital, Sun Yat-sen University 26469 , Guangzhou, China; 3 Laboratory of Mycology, Department of Dermatology, Huashan Hospital, Fudan University 12478, Shanghai, China; 4 Department of Clinical Laboratory, Union Hospital, Tongji Medical College, Huazhong University of Science and Technology 12443, Wuhan, China; 5 Department of Laboratory Medicine, Clinical Laboratory Medicine Research Center, West China Hospital, Sichuan University, Sichuan Clinical Research Center for Laboratory Medicine 617913, Chengdu, China; 6 Department of Clinical Laboratory, Provincial Hospital Affiliated to Fuzhou University 117861, Fuzhou, China; 7 Department of Laboratory Diagnostics, Changhai Hospital, Naval Military Medical University 12521, Shanghai, China; 8 Department of Clinical Laboratory, The Affiliated Hospital of Qingdao University 235960, Qingdao, China; 9 Department of Laboratory Medicine, The First Affiliated Hospital with Nanjing Medical University 74734 , Nanjing, China; 10 Branch of National Clinical Research Center for Laboratory Medicine, The First Affiliated Hospital with Nanjing Medical University 74734 , Nanjing, China; 11 Department of Laboratory Medicine, The First Affiliated Hospital of Guangzhou Medical University 26468, Guangzhou, China; 12 Clinical Biobank, National Infrastructures for Translational Medicine, Institute of Clinical Medicine, Peking Union Medical College Hospital, Chinese Academy of Medical Sciences and Peking Union Medical College 34732, Beijing, China; University Children's Hospital Münster, Münster, Germany

**Keywords:** invasive fungal diseases, antifungal resistance, surveillance, candidiasis

## Abstract

Invasive candidiasis (IC) is a severe infection primarily affecting immunocompromised patients and is associated with high mortality and substantial hospital costs. Accurate regional data on epidemiology and antifungal resistance of IC are crucial for effective clinical management. The China Hospital Invasive Fungal Surveillance Net (CHIF-NET) study is a laboratory-based multicenter study initiated in August 2009. This report presented updated data from August 2017 to December 2021, involving 76 hospitals across 28 provincial regions in China, and included a 12-year longitudinal analysis. Isolates from patients with IC were assigned to species level by matrix-assisted laser desorption ionization-time of flight mass spectrometry and internal transcribed spacer rDNA sequencing. Susceptibility testing was performed for nine antifungal agents using the broth microdilution method. A total of 11,679 *Candida* isolates were collected. *Candida albicans* was the most common *Candida* species (46.0%), followed by *Candida tropicalis* (17.6%), *Candida parapsilosis sensu stricto* (15.6%), and *Candida glabrata sensu stricto* (11.2%). Antifungal resistance rates showed notable variations among different *Candida* species, with the highest azole resistance observed in *C. tropicalis* isolates causing candidemia (42.5% to fluconazole and 38.9% to voriconazole). Cross- and multi-drug resistance to azoles and echinocandins was observed in all predominant *Candida* species. The species distribution of major *Candida* pathogens causing IC remained stable in China, while antifungal resistance increased, especially among non-*albicans Candida* species. Enhanced surveillance, accurate species identification, and strengthened antifungal stewardship are needed to address the growing challenge of antifungal resistance.

## INTRODUCTION

Invasive candidiasis (IC) is a severe infection that mainly affects immunocompromised patients, which is associated with high mortality and substantial additional hospital costs (1). Globally, the incidence of IC is increasing (2). Concurrently, the prevalence of antifungal resistance, both in *Candida albicans* and non-*albicans* species, as well as multi-drug resistance (e.g., in *Candida auris*), is also on the rise (3, 4). These trends are further complicated by the limited availability of effective antifungal therapeutic options, highlighting the urgent need for improved diagnostics, enhanced antifungal stewardship, and the development of novel antifungal agents (5). Given these challenges, accurate regional data on the epidemiology of IC and corresponding antifungal resistance patterns are crucial for guiding clinical practice and developing effective management strategies (6).

The distribution of *Candida* species causing IC and antifungal resistance varies significantly across different regions and demographic groups (7). The China Hospital Invasive Fungal Surveillance Net (CHIF-NET) study was a nationwide surveillance program initiated in 2009 (8). In previous studies, our team has reported the data from the first 5-year period (CHIF-NET 2010–2014) (9) and the subsequent 3-year period (CHIF-NET 2015–2017) (10). In this study, we present the most recent 4-year surveillance results on IC, covering the surveillance period CHIF-NET 2018–2021, to provide an updated view of the trends and characteristics of IC in China.

## MATERIALS AND METHODS

### Study design

The CHIF-NET study is a laboratory-based multicenter study of invasive yeast infections, which was initiated in August 2009 (8), and the inclusion criteria for isolates have been described previously (8–10). Of note, before CHIF-NET 2018, each surveillance year spanned from August of the preceding year to July of the current year (e.g., CHIF-NET 2010 covered August 2009 to July 2010). To facilitate data management and analysis, the surveillance period was extended in CHIF-NET 2018 to cover August 2017 through December 2018. Thereafter, each surveillance year was aligned with the calendar year (January–December). Accordingly, the data set analyzed in this study spans from August 2017 to December 2021, covering a total period of 4 years and 4 months. A total of 76 hospitals participated in the study during this period (with 45–55 hospitals included in each surveillance year), covering 28 provincial administrative regions of China. Each surveillance year, isolates from eligible patients with invasive fungal diseases were forwarded to the central laboratory (Department of Laboratory Medicine, Peking Union Medical College Hospital, Beijing, China) for confirmative species identification and antifungal susceptibility testing. Related processing procedures were as previously described (10).

### Species identification

All *Candida* isolates were identified to the species level in the central laboratory by matrix-assisted laser desorption ionization-time of flight mass spectrometry (MALDI-TOF MS) using Autof MS 1000 (Autobio Diagnostics, Zhengzhou, China) or Vitek MS (bioMérieux, Marcy l’Etoile, France) system.

For any isolates belonging to closely related yeast species complexes—including the *C. albicans* species complex (*C. albicans* and *Candida dubliniensis*), the *Candida parapsilosis* species complex (*C. parapsilosis sensu stricto*, *Candida metapsilosis*, *Candida orthopsilosis*, and *Lodderomyces elongisporus*), the *Candida glabrata* species complex (*C. glabrata sensu stricto*, *Candida nivariensis*, and *Candida bracarensis*), and the *Candida haemulonii-C. auris* species complex (*C. haemulonii sensu stricto*, *Candida duobushaemulonii*, *Candida pseudohaemulonii,* and *C. auris*), as well as for isolates with low-confidence MALDI-TOF MS results—sequencing of the internal transcribed spacer (ITS) rDNA region was performed for confirmatory species identification. The criteria for identification and discrepant analysis were as previously described (11).

### Antifungal susceptibility testing

Susceptibility testing was performed against nine antifungal agents using in-house-prepared 96-well microdilution plates: four azoles, that is, fluconazole (FZ), voriconazole (VOR), itraconazole (IZ), and posaconazole (PZ); three echinocandins, that is, caspofungin (CAS), micafungin (MF), and anidulafungin (AND); as well as 5-flucytosine (5-FC), and amphotericin B (AMB). The operating procedure and result interpretation were referred to the Clinical and Laboratory Standards Institute (CLSI) M27M44SED3 and M57SED4 documents (12, 13). For *C. albicans*, clinical breakpoints (CBPs) were applied to FZ, VOR, CAS, MF, and AND, and epidemiological cutoff values (ECVs) were used for PZ and AMB. For *C. parapsilosis sensu stricto*, *C. tropicalis*, and *Candida krusei*, CBPs were applied to FZ (except for *C. krusei*, which is intrinsically resistant to FZ), VOR, CAS, MF, and AND, and ECVs were used for IZ, PZ, and AMB. For *C. glabrata sensu stricto*, CBPs were applied to FZ, CAS, MF, and AND, whereas ECVs were used for VOR, IZ, PZ, and AMB. Quality control was performed using *C. krusei* ATCC 6258 and *C. parapsilosis* ATCC 22019. Isolates that were resistant (R) or non-wild-type (NWT) to ≥2 agents within the same antifungal class were considered cross-resistant, whereas isolates that were R or NWT to agents from at least two of the following classes—azoles, echinocandins, and amphotericin B—were considered multi-drug resistant (5-FC was not included due to the lack of CBPs or ECVs).

### Statistical analysis

Statistical analyses and data visualization were performed using R (Version 4.2.1) (https://cran.r-project.org). Comparisons of categorical variables were performed using the χ² test or Fisher’s exact test, as appropriate. A *P*-value <0.05 was considered statistically significant.

## RESULTS

### Overall species distribution

A total of 11,679 non-repetitive *Candida* isolates were collected in CHIF-NET 2018–2021 (Table 1), which accounted for 90.2% of all 12,954 yeast isolates identified during this period, and 37.0% (4,325/11,679) of the *Candida* isolates were isolated from female patients. The age of patients ranged from 0 to 104 years old (median: 60, interquartile range [IQR]: 24–71). Among all *Candida* isolates, *C. albicans* was the most common species overall and within each surveillance year, accounting for 46.0% (5,368/11,679) of the isolates in total. *C. tropicalis* and *C. parapsilosis sensu stricto* were the second and third most common species, accounting for 17.6% (2,054/11,679) and 15.6% (1,821/11,679) of the collection, followed by *C. glabrata sensu stricto* (1,306/11,679, 11.2%). Within the *C. haemulonii–C. auris* species complex (*n* = 34), which is of global concern, *C. haemulonii sensu stricto* was the most common (*n* = 21), followed by *C. duobushaemulonii* (*n* = 7), *C. auris* (*n* = 5), and *C. pseudohaemulonii* (*n* = 1). Other *Candida* species with a prevalence of >1% included *C. metapsilosis* (235/11,679, 2.0%), *C. krusei* (213/11,679, 1.8%), *C. orthopsilosis* (165/11,679, 1.4%), *C. guilliermondii* (149/11,679, 1.3%), and *C. lusitaniae* (118/11,679, 1.0%), and the rest rare *Candida* species added up to 1.8% (216/11,679).

**TABLE 1:** Distribution of *Candida* species in CHIF-NET 2018–2021

Species/species complex	Overall	CHIF-NET 2018	CHIF-NET 2019	CHIF-NET 2020	CHIF-NET 2021
*C. albicans* species complex	5,374 (46.0%)	1,557 (43.2%)	1,289 (48.7%)	1,068 (47.7%)	1,460 (45.8%)
*C. albicans*	5,368 (46.0%)	1,556 (43.2%)	1,287 (48.6%)	1,067 (47.7%)	1,458 (45.7%)
*C. dubliniensis*	6 (0.1%)	1 (0.0%)	2 (0.1%)	1 (0.0%)	2 (0.1%)
*C. parapsilosis* species complex	2,255 (19.3%)	695 (19.3%)	448 (16.9%)	441 (19.7%)	671 (21.1%)
*C. parapsilosis sensu stricto*	1,821 (15.6%)	556 (15.4%)	338 (12.8%)	363 (16.2%)	564 (17.7%)
*C. metapsilosis*	235 (2.0%)	73 (2.0%)	58 (2.2%)	44 (2.0%)	60 (1.9%)
*C. orthopsilosis*	165 (1.4%)	53 (1.5%)	46 (1.7%)	29 (1.3%)	37 (1.2%)
*Lodderomyces elongisporus*	34 (0.3%)	13 (0.4%)	6 (0.2%)	5 (0.2%)	10 (0.3%)
*C. tropicalis*	2,054 (17.6%)	731 (20.3%)	429 (16.2%)	393 (17.6%)	501 (15.7%)
*C. glabrata* species complex	1,320 (11.3%)	415 (11.5%)	318 (12.0%)	226 (10.1%)	361 (11.3%)
*C. glabrata sensu stricto*	1,306 (11.2%)	412 (11.4%)	317 (12.0%)	221 (9.9%)	356 (11.2%)
*C. nivariensis*	11 (0.1%)	2 (0.1%)	1 (0.0%)	4 (0.2%)	4 (0.1%)
*C. bracarensis*	3 (0.0%)	1 (0.0%)	0 (0.0%)	1 (0.0%)	1 (0.0%)
*C. krusei*	213 (1.8%)	69 (1.9%)	63 (2.4%)	28 (1.3%)	53 (1.7%)
*C. guilliermondii*	149 (1.3%)	32 (0.9%)	40 (1.5%)	28 (1.3%)	49 (1.5%)
*C. lusitaniae*	118 (1.0%)	21 (0.6%)	31 (1.2%)	22 (1.0%)	44 (1.4%)
*C. pelliculosa*	53 (0.5%)	33 (0.9%)	6 (0.2%)	7 (0.3%)	7 (0.2%)
*C. haemulonii-C. auris* species complex	34 (0.3%)	18 (0.5%)	6 (0.2%)	6 (0.3%)	4 (0.1%)
*C. haemulonii sensu stricto*	21 (0.2%)	10 (0.3%)	5 (0.2%)	4 (0.2%)	2 (0.1%)
*C. duobushaemulonii*	7 (0.1%)	3 (0.1%)	1 (0.0%)	1 (0.0%)	2 (0.1%)
*C. auris*	5 (0.0%)	5 (0.1%)	0 (0.0%)	0 (0.0%)	0 (0.0%)
*C. pseudohaemulonii*	1 (0.0%)	0 (0.0%)	0 (0.0%)	1 (0.0%)	0 (0.0%)
*C. fabianii*	27 (0.2%)	7 (0.2%)	8 (0.3%)	3 (0.1%)	9 (0.3%)
*C. norvegensis*	27 (0.2%)	8 (0.2%)	4 (0.2%)	7 (0.3%)	8 (0.3%)
*C. rugosa*	13 (0.1%)	6 (0.2%)	0 (0.0%)	2 (0.1%)	5 (0.2%)
*C. kefyr*	9 (0.1%)	2 (0.1%)	2 (0.1%)	1 (0.0%)	4 (0.1%)
Other *Candida* species	33 (0.3%)	12 (0.3%)	4 (0.2%)	6 (0.3%)	11 (0.3%)
Overall	11,679 (100.0%)	3,606 (100.0%)	2,648 (100.0%)	2,238 (100.0%)	3,187 (100.0%)

### Hospital departments and specimen types

**Fig 1:**
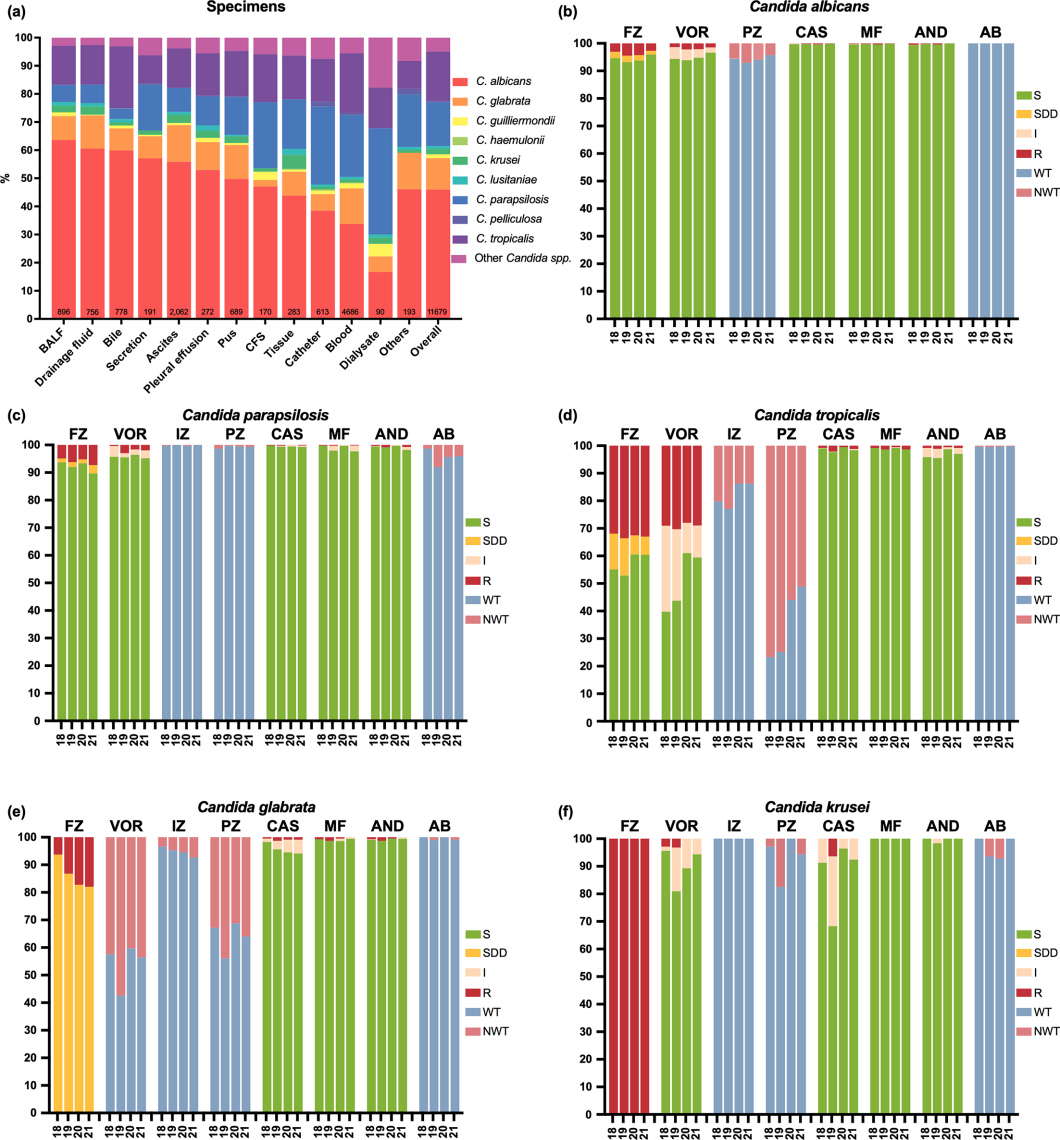
Overall species distribution and antifungal susceptibility profiles in all invasive candidiasis cases. (**a**) Species distribution by specimen type. The total number of each specimen type is indicated at the bottom of the bar chart. (**b**) Antifungal susceptibility profile of *C. albicans,* (**c**) *C. parapsilosis*, (**d**) *C. tropicalis,* (**e**) *C. glabrata*, and (**f**) *C. krusei*. Abbreviations: BALF, bronchoalveolar lavage fluid; S, susceptible; SDD, susceptible-dose dependent; I, intermediate; R, resistant; WT, wild type; NWT, non-wild type.

This study included not only *Candida* isolates from bloodstream infections (4,686/11,679, 40.1%) but also those causing other types of IC. Among non-candidemia isolates, the most common source was ascites (2,062/11,679, 17.7%), followed by bronchoalveolar lavage fluid (BALF, 896/11,679, 7.7%), bile (778/11,679, 6.7%), drainage fluid (756/11,679, 6.5%), and pus (689/11,679, 5.9%). *C. albicans* was the most frequently isolated species among most specimen types, although its prevalence varied by source (Fig. 1a). In candidemia cases, a lower proportion of *C. albicans* (1,582/4,686, 33.8%) and a higher proportion of *C. parapsilosis sensu stricto* (1,030/4,686, 22.0%) were recognized. Relatively low proportions of *C. albicans* are also observed in dialysate (15/90, 16.7%).

**Fig 2:**
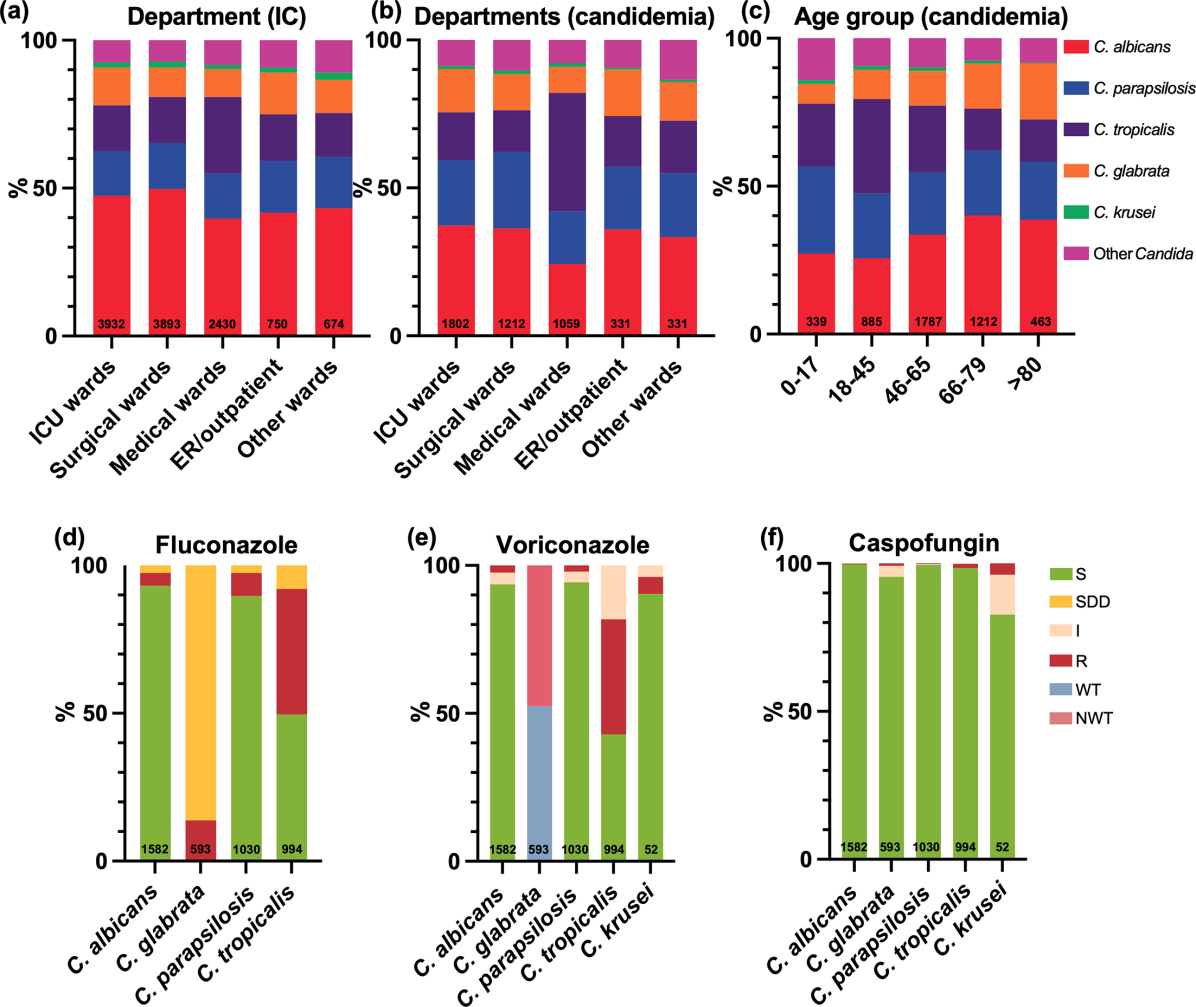
Distribution and antifungal susceptibility of the most prevalent *Candida* species in candidemia cases. (**a**) Ward distribution of major *Candida* species among all invasive candidiasis cases (as references for comparison). (**b**) Ward distribution of major *Candida* species in candidemia cases. (**c**) Age group distribution of major *Candida* species in candidemia cases. (**d**) Fluconazole susceptibility profile of major *Candida* species in candidemia. (**e**) Voriconazole susceptibility profile of major *Candida* species in candidemia. (**f**) Caspofungin susceptibility profile of major *Candida* species in candidemia. Abbreviations: ER, emergency room.

Figure 2a summarizes the distribution of *Candida* species across different clinical settings. Of all 11,679 isolates, 93.6% were from hospitalized patients, with the majority originating from intensive care units (ICUs, 33.7%), surgical wards (33.3%), medical wards (20.8%), and other hospitalized patients (5.8%). The remaining isolates were from outpatient (1.5%) or emergency (4.9%) settings. *C. albicans* was the most common species in all departments. However, *C. tropicalis* exhibited a relatively higher isolation rate in medical wards (627/2,430, 25.8%, *P* < 0.001), and the isolation rate of *C. albicans* in medical wards was the lowest (964/2,430, 39.7%) compared to their distribution in other departments (*P* < 0.001).

### *In vitro* susceptibility to azoles

The resistance rates, MIC_50_, MIC_90_, and geometric mean (GM) MIC values for all *Candida* species over the 4-year period are provided in the Supplementary Information, and the antifungal susceptibility profiles of predominant *Candida* species are shown in Fig. 1b through f. The four species within the *C. parapsilosis* species complex exhibited distinct azole susceptibility profiles. Of note, the resistance rates for FZ in *C. metapsilosis* and *C. orthopsilosis* increased over the 4-year period: for *C. orthopsilosis*, the FZ resistance rate increased from 35.8% in the first year to 56.8%–62.1% in the third and fourth years; and for *C. metapsilosis*, the FZ resistance rate increased from 3.4%–4.5% in the first 3 years to 10.0% in the fourth year. In contrast, FZ resistance rates in *C. albicans* and *C. tropicalis* remained relatively stable over the study period: consistently low in *C. albicans* (<5%) and persistently high in *C. tropicalis* (>30%). For *C. glabrata sensu stricto*, FZ resistance increased from 6.3% (26/412) in CHIF-NET 2018 to 18.0% (64/356) in CHIF-NET 2021. Compared with FZ, VOR generally exhibited lower resistance among major *Candida* species. Specifically, resistance to VOR was markedly lower than to FZ in *C. albicans* (1.7% vs. 3.5%, *P* < 0.001) and *C. parapsilosis sensu stricto* (1.6% vs. 5.9%, *P* < 0.001). Of *C. krusei* isolates, which were intrinsically resistant to FZ, 1.9% (4/213) were VOR-resistant and 8.0% (17/213) were intermediate. Besides, *C. tropicalis* showed similar resistance rates to VOR and FZ (29.1% vs. 32.6%, *P* = 0.015). Moreover, *C. glabrata sensu stricto* isolates exhibited high NWT rates to VOR, ranging from 40.3% to 57.4% over the 4-year period. For IZ, almost all (>99%) *C. parapsilosis sensu stricto* and *C. krusei* isolates were of WT phenotypes. In addition, no *C. krusei* isolates were NWT to IZ. The prevalence of *C. glabrata sensu stricto* isolates of NWT phenotype to IZ stayed below 10%, but rose gradually from 3.4% (14/412) in CHIF-NET 2018 to 7.3% (26/356) in CHIF-NET 2021. *C. tropicalis* showed the highest NWT rates to IZ, exceeding 20% in CHIF-NET 2019 (98/429, 22.8%) and subsequently declining to 13.7% (54/393) in CHIF-NET 2020. In comparison, the NWT rates against PZ were high among all common *Candida* species except for *C. parapsilosis sensu stricto*, which was around 5% in *C. albicans* and *C. krusei*, 65% in *C. tropicalis*, and >30% in *C. glabrata sensu stricto*. Only five *C. auris* isolates were identified in CHIF-NET 2018, all of which originated from the same hospital and were 100% resistant to FZ.

### *In vitro* susceptibility to echinocandins, amphotericin B, and 5-flucytosine

For the echinocandins, susceptibility testing was performed for three drugs: CAS, MF, and AND. The results are presented in Fig. 1b through f. All three echinocandin agents demonstrated good *in vitro* activity against common *Candida* species. *C. albicans* and *C. parapsilosis sensu stricto* were ≥99% susceptible to CAS, MF, and AND. Over 98% of *C. tropicalis* isolates were susceptible to CAS and MF, and the resistance rate to AND was also <1%, but 2.5% isolates were intermediate to AND. *C. glabrata sensu stricto* and *C. krusei* isolates were also >99% susceptible to MF and AND. However, a 6.3% CAS resistance rate (4/63) was observed in *C. krusei* in CHIF-NET 2019, and the proportion of *C. krusei* isolates intermediate to CAS fluctuated between 3.6% and 25.4% over the four surveillance years. Besides, CAS intermediate rates in *C. glabrata sensu stricto* increased gradually from 1.2% to 5.1% over the 4-year period.

For AMB, over 99% isolates were of WT phenotype in *C. albicans* (5,365/5,368, 99.9%), *C. tropicalis* (2,047/2,054, 99.7%), and *C. glabrata sensu stricto* (1,300/1,306, 99.5%), while 4.0% *C. parapsilosis sensu stricto* (73/1,821) and 2.8% *C. krusei* (6/213) isolates were NWT. The MIC_50_ values of 5-FC for *C. albicans*, *C. tropicalis*, *C. glabrata sensu stricto*, and *C. parapsilosis sensu stricto* remained consistently low (0.06–0.12 μg/mL) over the surveillance period. In contrast, *C. krusei* exhibited higher 5-FC MIC_50_ values, ranging from 8 to 16 μg/mL (Table S1).

### Candidemia

Among the 4,686 candidemia isolates, species distribution in surgical wards and outpatient/emergency settings was generally consistent with the overall IC distribution in these departments (Fig. 2b). However, notable discrepancies in species distribution between candidemia and overall IC were observed in the ICU and internal medicine wards. In the ICU wards, although *C. albicans* remained the most common species causing candidemia, its proportion (672/1,802, 37.3%) was lower than that observed among all IC cases in this setting (1,865/3,932, 47.4%). Meanwhile, *C. parapsilosis sensu stricto* ranked second (398/1,802, 22.1%), exceeding *C. tropicalis* (291/1,802, 16.1%), which also differed from the species ranking observed in overall IC cases in the ICU. In medical wards, *C. tropicalis* has become the leading cause of candidemia (425/1,059, 40.1%), surpassing *C. albicans* (257/1,059, 24.3%) and *C. parapsilosis sensu stricto* (188/1,059, 17.8%).

Among different age groups, patients aged 46–65 years accounted for the highest proportion of candidemia isolates (1,787/4,686, 38.1%) (Fig. 2c). The distribution of *Candida* species and the proportional incidence of candidemia across different age groups remained consistent with previous findings in CHIF-NET 2015–2017 (10). The antifungal susceptibility of the five most common *Candida* species isolated from blood to FZ, VOR, and CAS is shown in Fig. 2c through f. *C. tropicalis* exhibited the most notable azole resistance, and its overall susceptibility rate was only 49.6% (493/994) to FZ and 42.9% (426/994) to VOR over the 4-year period. Corresponding resistance rates of *C. tropicalis* to FZ and VOR were 42.5% (422/994) and 38.9% (387/994), respectively. The susceptibility rates had a further decline, compared to the previous reporting period (63.5% and 49.2%, respectively) (10). For CAS, the highest resistance rate was observed in *C. krusei* (2/52, 3.8%), followed by *C. tropicalis* (14/994, 1.4%). Resistance rates to CAS in *C. albicans*, *C. glabrata sensu stricto*, and *C. parapsilosis sensu stricto* remained below 1%. For FZ, VOR, and CAS, susceptibility rates were generally lower in candidemia isolates than in the overall IC population.

### Cross-resistance and multi-drug resistance to azoles or echinocandins

We analyzed the prevalence of cross-resistance and multi-drug resistance across the four tested azoles (FZ, VOR, IZ, and PZ), three echinocandins (CAS, MF, and AND), and amphotericin B (AMB). The results are shown in Table 2. Among the azoles, *C. glabrata sensu stricto* had the highest rate of azole cross-resistance, with 34.9% of strains resistant to at least two azole drugs. Besides, 17.0% of *C. tropicalis* strains are resistant to all four azole drugs. Although overall resistance rates to echinocandins were low, isolates that are cross-resistant to all three echinocandins and multi-drug resistant have been identified in most of the common *Candida* species including *C. albicans*, *C. tropicalis*, *C. parapsilosis sensu stricto,* and *C. glabrata sensu stricto*.

**TABLE 2:** Cross- and multi-drug resistance in CHIF-NET 2018–2021

Species	Cross-resistance to azoles (%)^*a*^			Cross-resistance to echinocandins (%)		Multi-drug resistance (%)
	2	3	4	2	3	
*C. albicans* (*n* = 5,368)	1.7	1.6	0.0	0.1	0.1	0.1
*C. tropicalis* (*n* = 2,054)	3.9	12.0	17.0	0.2	0.8	1.1
*C. parapsilosis sensu stricto* (*n* = 1,821)	2.0	0.1	0.1	0.0	0.2	1.0
*C. glabrata sensu stricto* (*n* = 1,306)	22.5	7.4	5.0	0.1	0.5	1.1
*C. metapsilosis* (*n* = 235)	0.9	0.0	0.4	0.0	0.4	1.7
*C. krusei* (*n* = 213)^*b*^	1.4	0.0	0.0	0.0	0.0	2.3
*C. orthopsilosis* (*n* = 165)	23.0	6.1	0.6	0.0	0.0	1.2

^*a*^ Cross-resistance: R or NWT to ≥2 agents within the same antifungal class (e.g., resistance to 2, 3, or 4 azole drugs).

^*b*^ Because *C. krusei* is intrinsically resistant to FZ, FZ was not included in cross-resistance and multi-drug resistance analyses for this species.

### Trends for antifungal susceptibility of major IC species over 12 years

**Fig 3:**
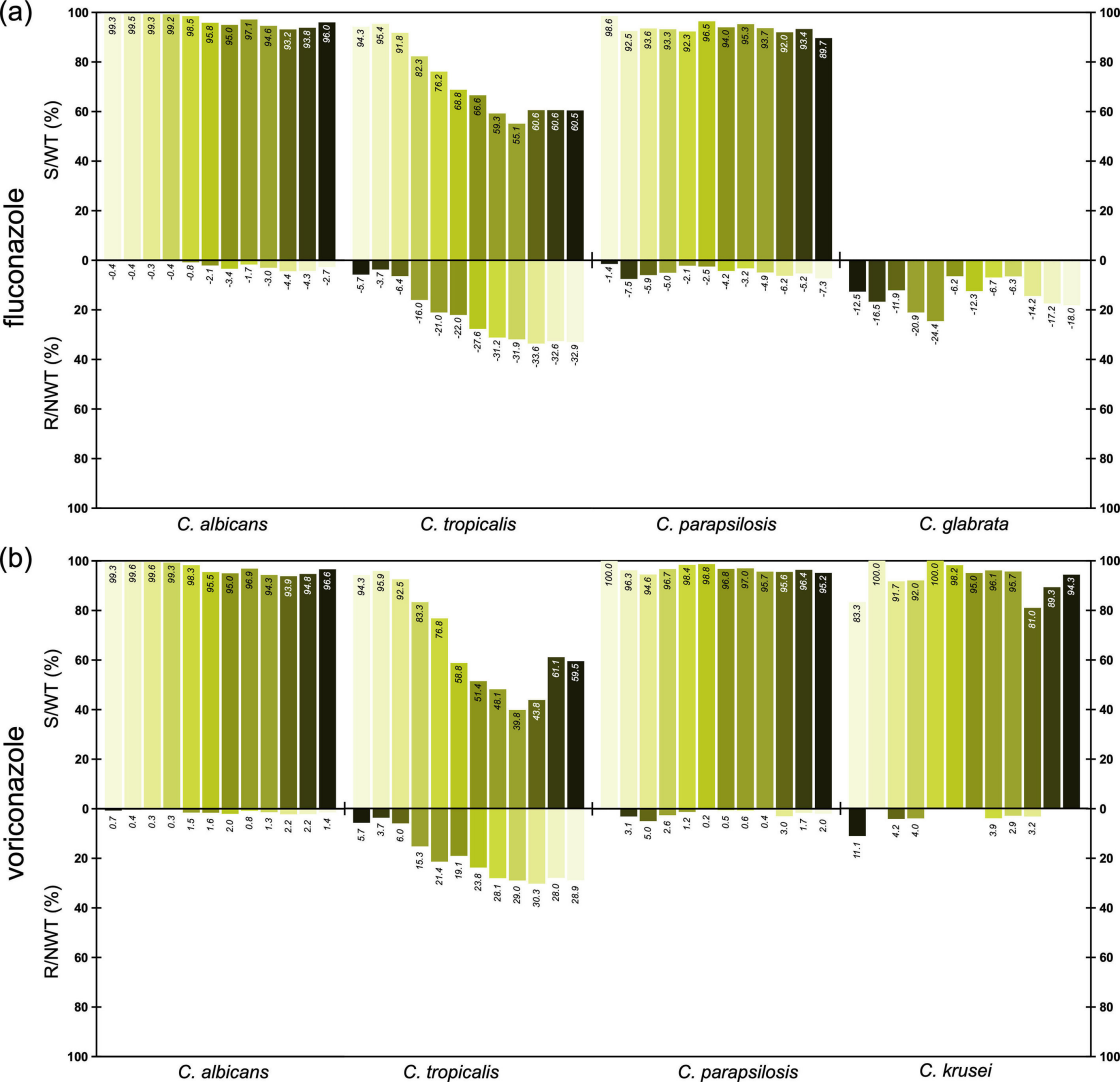
Trends in antifungal susceptibility among major *Candida* species causing invasive candidiasis from CHIF-NET 2010–2021. (**a**) Fluconazole. (**b**) Voriconazole. Annual data are presented from left to right, starting in CHIF-NET 2010 and ending in CHIF-NET 2021. Abbreviations: S, susceptible; R, resistant; WT, wild type; NWT, non-wild type.

We conducted a longitudinal comparison of the resistance trends of FZ and VOR (Fig. 3) against key *Candida* species over the 12 years since the establishment of CHIF-NET, including *C. albicans*, *C. tropicalis*, *C. parapsilosis sensu stricto*, *C. glabrata sensu stricto*, and *C. krusei*. In general, FZ susceptibility remained stable in *C. albicans* and *C. parapsilosis sensu stricto* (susceptibility rates >90%). *C. glabrata sensu stricto* exhibited increasing FZ resistance in CHIF-NET 2010–2014 (from 11.9% to 24.4%), followed by a sharp decline (6.2%) in CHIF-NET 2015 and a recent gradual rise to 18.0%. FZ resistance in *C. tropicalis* increased from <10% during CHIF-NET 2010–2012 to 34.5% in CHIF-NET 2019, accompanied by a decline in susceptibility from 94.3% to 51.7%. Both trends partially reversed in CHIF-NET 2020–2021, with FZ susceptibility rates rebounding to approximately 60%. For VOR, susceptibility rates for *C. albicans* and *C. parapsilosis sensu stricto* remained above 95%, with only a few exceptions. The longitude VOR susceptibility trend in *C. tropicalis* was similar to that of FZ. Susceptibility remained high (>92%) during CHIF-NET 2010–2012 but declined sharply thereafter, reaching a lowest level of 39.8% in CHIF-NET 2018, and began to reverse and rebound to approximately 60% in CHIF-NET 2020–2021. MF susceptibility testing for major *Candida* species causing invasive candidiasis has been conducted since CHIF-NET 2015. As shown in Fig. S1, resistance rates to MF remained consistently low over the seven-year monitoring period (CHIF-NET 2015–2021).

## DISCUSSION

Over the 12-year period since the initiation of the CHIF-NET study, the isolation rates of common *Candida* species have remained relatively stable, and the top five species have consistently been *C. albicans*, *C. parapsilosis* species complex, *C. tropicalis*, *C. glabrata* species complex, and *C. krusei*.

In 2022, the World Health Organization developed the first fungal priority pathogens list (WHO FPPL) (14). Both *C. auris* and *C. albicans* were included in the Critical Priority Group. *C. auris* (now classified as *Clavispora auris*), in particular, has garnered global attention in recent years due to its potential for rapid transmission and multi-drug resistance. Recent data from the SENTRY Antifungal Surveillance Program highlight a notable increase in the prevalence of *C. auris* isolates, rising from <0.1% prior to 2018 to 1.6% in 2022 (15). However, in the CHIF-NET surveillance program, only five *C. auris* were isolated in 2018, all from the same hospital, which suggests a potential localized outbreak. Nevertheless, the emergence and nosocomial transmission of *C. auris* in China have been documented in other more recent studies. A national retrospective survey conducted from January 2016 to August 2023 identified a total of 312 cases from 18 hospitals across 10 provinces, with a marked rise observed after 2021 (16). Notably, Guangdong Province in South China experienced an explosive outbreak, underscoring the ongoing challenges posed by this pathogen (16).

*C. albicans* remains the predominant causative agent of IC globally, while a number of studies have reported an increasing trend in non-*albicans Candida* species (17, 18). Notably, the proportion of *C. albicans* among candidemia cases in our study was only 33.8% (1,582/4,686), which is lower than that reported in the UK (accounting for 44.8% in 2020) (19) and the US (where the proportion declined from 52% in 1992–1993 to 41% in 2008–2011) (20). This underscores the substantial clinical challenge posed by non-*albicans Candida* species in China.

The other four most prevalent *Candida* species, i.e., *C. parapsilosis*, *C. tropicalis*, *C. glabrata*, and *C. krusei*, were listed in the High Priority Group of the WHO FPPL. *C. parapsilosis*, as the most prevalent non-*albicans Candida* species, exhibits a high isolation rate. *C. parapsilosis* infections have become particularly concerning in the past few years due to a strong rise in azole resistance and a number of fluconazole-resistant *C. parapsilosis* nosocomial outbreaks (21–23). Of note, species within the *C. parapsilosis* complex differed in isolation frequency and exhibited distinct antifungal resistance profiles, underscoring the importance of accurate species-level identification for guiding antifungal therapy (24). Notably, *C. orthopsilosis* displayed markedly higher FZ resistance in our study and reached 62.1% in CHIF-NET 2020. In contrast, *C. metapsilosis* and *C. parapsilosis sensu stricto* were less resistant (FZ resistance rate around 5%). However, in CHIF-NET 2021, a notable increase in fluconazole resistance was observed in both *C. metapsilosis* and *C. parapsilosis sensu stricto*. Additionally, *C. parapsilosis* can persist in hospital environments, and the recently recognized phenomenon of antifungal heteroresistance poses a new clinical challenge (25).

*C. tropicalis*, known for its high prevalence in tropical geographic regions (26), is the most common non-*albicans Candida* species causing candidemia in India, Thailand, and Singapore (27). The 30-day mortality of candidemia caused by *C. tropicalis* has been reported to be up to 52% (28). In this study, the prevalence of *C. tropicalis* was notably higher in medical wards among candidemia cases. Besides, *C. tropicalis* exhibits the highest rates of azole resistance among all common *Candida* species in China. Notably, fluconazole resistance rates varied across the world, typically ranging from 0% to 18% (29). Fluconazole remained one of the cost-effective first-line antifungal agents for prophylaxis against IC in hematopoietic stem cell transplant recipients (30, 31). Primary fluconazole resistance is relatively uncommon in *C. tropicalis*; however, resistance may develop upon drug exposure (32), and extensive azole use in clinical practice may contribute to the increasing resistance observed in this species (33). However, a recent study demonstrated that agricultural triazole fungicides can also induce cross-resistance to clinical azole drugs in *C. tropicalis* because these compounds share the same molecular target (34). Similar concerns regarding agriculture-driven azole resistance have previously been recognized in *Aspergillus fumigatus* (35). Therefore, coordinated efforts across human, animal, and environmental sectors are needed to reduce the risk of cross-sector antimicrobial resistance.

In our study, *C. glabrata sensu stricto* (now classified as *Nakaseomyces glabrata*) accounted for 11.2% of IC and 12.7% of candidemia cases, ranking fourth in both groups. In Japan, *C. glabrata* has been reported as the predominant non-*albicans Candida* species, comprising 19.5% of candidemia isolates (36). In comparison, surveillance data from the UK indicate that *C. glabrata* remained the most frequently isolated non-*albicans Candida* species, accounting for 29.4% and 25.5% of candidemia episodes in 2019 and 2020, respectively (19). The SENTRY Program (1997–2016) reported a *C. glabrata* isolation rate of 24.3% among candidemia in North America (37). These results indicated regional variations in the epidemiology of this species. Moreover, antifungal susceptibility in *C. glabrata* also varies considerably across regions (38). In the US, micafungin resistance in *C. glabrata* increased from negligible levels during 2001–2007 to over 6% in *sensu stricto* 2014–2016 (37), while in Japan, resistance rates ranged from 8.0% to 17.8% in a study spanning 2010–2019 (36). Notably, about 10% of clinical *C. glabrata* isolates have been reported to exhibit multi-drug resistance to both azole and echinocandin agents (39, 40). In contrast, the echinocandin resistance in China remained low and <1% in *C. glabrata* till CHIF-NET 2021. Alarmingly, among echinocandin-resistant *C. glabrata* isolates, around 36%–38% also exhibited fluconazole resistance (39). However, in our study, the overall multi-drug resistance rate in *C. glabrata* was 1.1%, and only 0.6% of isolates exhibited resistance to both azoles and echinocandins. However, it should be noted that echinocandin use has increased substantially in China in recent years, underscoring the need for continued surveillance to monitor emerging resistance trends among *C. glabrata* and other *Candida* species.

Indeed, over the 12 years of CHIF-NET surveillance (2010–2021), notable changes in antifungal resistance were observed. The most notable change was the marked decline in susceptibility of *C. tropicalis* to both FZ and VOR, with susceptibility rates decreasing from over 90% in the early surveillance period to approximately 50%. The observed increase in resistance among *C. tropicalis* isolates in China is largely attributed to the clonal dissemination of strains exhibiting novel antifungal resistance mechanisms within the healthcare settings (41, 42). While fluconazole resistance remained relatively low in *C. albicans* and *C. parapsilosis*, consistent shifts in susceptibility were observed, highlighting the importance of ongoing antifungal resistance surveillance.

The limitations of our study remained. As a laboratory-based study, our data lack detailed clinical information. Additionally, our data are compiled biannually, and the interval from isolate transportation to completion of analysis takes approximately 8–10 months. Consequently, surveillance results may lag behind the most recent epidemiological developments, such as the rapid spread of *C. auris* in recent years. Efforts are ongoing to further improve the timeliness and completeness of the surveillance system.

### Conclusion

IC poses a significant global health threat, as the rising antifungal resistance increasingly complicates treatment and infection control worldwide. This study provides updated data on the epidemiology and antifungal susceptibility of IC pathogens in China. We observed high resistance rates, particularly among non-*albicans Candida* species such as *C. tropicalis*, along with the emergence of multi-drug resistance. These findings support more informed empirical therapy and antifungal stewardship efforts and highlight the need for continued surveillance and targeted clinical management to reduce the burden of IC in China and beyond.

## Data Availability

The data that support the findings of this study are available on request from the corresponding author. The data are not publicly available due to privacy or ethical restrictions.

## SUPPLEMENTAL MATERIAL

Supplemental material aac.00035-26-s0001.docx Fig. S1; Table S1.

ASM does not own the copyrights to Supplemental Material that may be linked to, or accessed through, an article. The authors have granted ASM a non-exclusive, world-wide license to publish the Supplemental Material files. Please contact the corresponding author directly for reuse.
