# Supplementary material for: Surveillance for invasive candidiasis in China (CHIF-NET 2018-2021): rising antifungal resistance observed in a nationwide longitudinal study
Source: Antimicrob Agents Chemother. 2026 Apr 27;70(6):e00035-26. doi: 10.1128/aac.00035-26 (PMC13231925; doi:10.1128/aac.00035-26)
Supplement: Supplemental material — Fig. S1; Table S1. [file aac.00035-26-s0001.docx]

Supporting Information for

Surveillance for Invasive Candidiasis in China (CHIF-NET 2018-2021): Rising Antifungal Resistance Observed in a Nationwide Longitudinal Study

**Qiao-Lian Yi, et al.**

**Corresponding author: Ying-Chun Xu and Meng Xiao**

**cjtcxiaomeng@aliyun.com (M. X.) and xycpumch@139.com (Y.-C. X.)**

The file includes:

Figure S1. Trend of antifungal susceptibility of micafungin for major Invasive Candidiasis species over 7 years.

Table S1. MIC_50_, MIC_90_, and geometric mean for major *Candida* species.

Other Supplementary Material for this manuscript includes the following are available from the corresponding author with reasonable request.

**
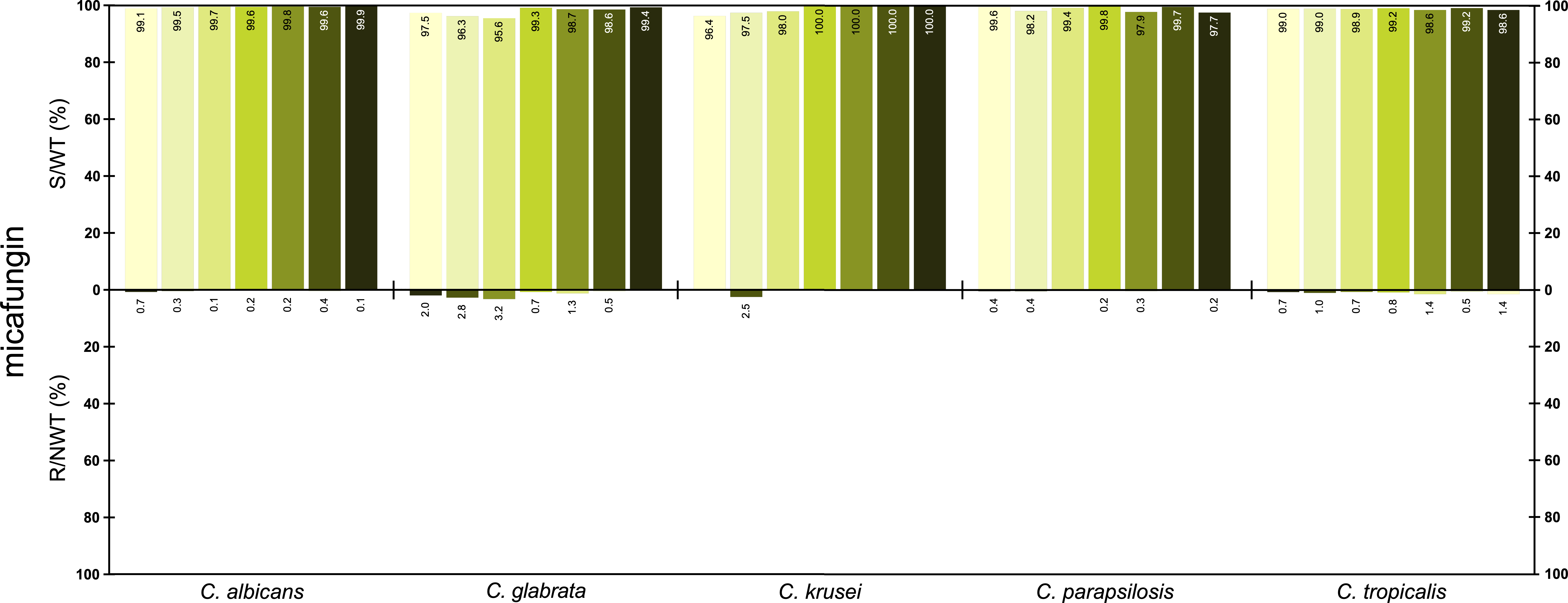
**

**Figure S1** Trend of antifungal susceptibility of micafungin for major Invasive Candidiasis species over 7 years. Annual data are presented from left to right, starting from 2015 and ending in 2021. (S: susceptible; R: resistant; WT: wide tpye; NWT: non-wide type).

**Table S1** MIC_50_, MIC_90_, and geometric mean for major *Candida* species.

| Species | fluconazole | | |  | voriconazole | | |  | itraconazole | | |  | posaconazole | | |  | caspofungin | | |  | anidulafungin | | |  | amphotericin B | | |  | 5-flucytosine | | |
| --- | --- | --- | --- | --- | --- | --- | --- | --- | --- | --- | --- | --- | --- | --- | --- | --- | --- | --- | --- | --- | --- | --- | --- | --- | --- | --- | --- | --- | --- | --- | --- |
|  | MIC_50_ | MIC_90_ | GM |  | MIC_50_ | MIC_90_ | GM |  | MIC_50_ | MIC_90_ | GM |  | MIC_50_ | MIC_90_ | GM |  | MIC_50_ | MIC_90_ | GM |  | MIC_50_ | MIC_90_ | GM |  | MIC_50_ | MIC_90_ | GM |  | MIC_50_ | MIC_90_ | GM |
| *C. albicans* | | | | | | | | | | | | | | | | | | | | | | | | | | | | | | | |
| CHIF-NET18 | 0.5 | 1 | 0.48 |  | 0.008 | 0.03 | 0.01 |  | 0.06 | 0.12 | 0.06 |  | 0.03 | 0.06 | 0.03 |  | 0.03 | 0.06 | 0.03 |  | 0.03 | 0.12 | 0.03 |  | 0.5 | 0.5 | 0.46 |  | 0.06 | 0.12 | 0.09 |
| CHIF-NET19 | 0.5 | 1 | 0.61 |  | 0.008 | 0.03 | 0.01 |  | 0.06 | 0.12 | 0.08 |  | 0.03 | 0.06 | 0.04 |  | 0.03 | 0.06 | 0.04 |  | 0.03 | 0.12 | 0.03 |  | 0.5 | 1 | 0.50 |  | 0.06 | 0.12 | 0.09 |
| CHIF-NET20 | 0.5 | 2 | 0.66 |  | 0.008 | 0.03 | 0.01 |  | 0.06 | 0.12 | 0.05 |  | 0.03 | 0.06 | 0.03 |  | 0.03 | 0.06 | 0.04 |  | 0.015 | 0.06 | 0.02 |  | 0.5 | 1 | 0.46 |  | 0.06 | 0.12 | 0.08 |
| CHIF-NET21 | 0.5 | 1 | 0.63 |  | 0.008 | 0.03 | 0.01 |  | 0.06 | 0.12 | 0.05 |  | 0.03 | 0.06 | 0.03 |  | 0.03 | 0.06 | 0.04 |  | 0.015 | 0.06 | 0.02 |  | 0.5 | 1 | 0.45 |  | 0.06 | 0.12 | 0.09 |
| CHIF-NET18-21 | 0.5 | 1 | 0.59 |  | 0.008 | 0.03 | 0.01 |  | 0.06 | 0.12 | 0.06 |  | 0.03 | 0.06 | 0.03 |  | 0.03 | 0.06 | 0.04 |  | 0.015 | 0.12 | 0.03 |  | 0.5 | 1 | 0.47 |  | 0.06 | 0.12 | 0.09 |
| *C. parapsilosis* complex | | | | | | | | | | | | | | | | | | | | | | | | | | | | | | | |
| CHIF-NET18 | 0.5 | 2 | 0.78 |  | 0.015 | 0.06 | 0.02 |  | 0.06 | 0.12 | 0.07 |  | 0.06 | 0.12 | 0.05 |  | 0.5 | 1 | 0.41 |  | 1 | 2 | 0.77 |  | 0.5 | 1 | 0.50 |  | 0.06 | 0.12 | 0.10 |
| CHIF-NET19 | 1 | 4 | 1.10 |  | 0.015 | 0.06 | 0.02 |  | 0.12 | 0.25 | 0.09 |  | 0.06 | 0.12 | 0.06 |  | 1 | 1 | 0.58 |  | 1 | 2 | 0.79 |  | 0.5 | 1 | 0.57 |  | 0.06 | 0.25 | 0.12 |
| CHIF-NET20 | 0.5 | 4 | 0.87 |  | 0.015 | 0.06 | 0.02 |  | 0.06 | 0.12 | 0.05 |  | 0.03 | 0.12 | 0.04 |  | 0.5 | 1 | 0.43 |  | 1 | 2 | 0.61 |  | 0.5 | 1 | 0.47 |  | 0.06 | 0.25 | 0.10 |
| CHIF-NET21 | 0.5 | 8 | 0.94 |  | 0.015 | 0.12 | 0.02 |  | 0.06 | 0.12 | 0.05 |  | 0.03 | 0.12 | 0.04 |  | 0.5 | 1 | 0.48 |  | 1 | 2 | 0.68 |  | 0.5 | 1 | 0.50 |  | 0.06 | 0.12 | 0.10 |
| CHIF-NET18-21 | 0.5 | 4 | 0.90 |  | 0.015 | 0.06 | 0.02 |  | 0.06 | 0.12 | 0.06 |  | 0.06 | 0.12 | 0.05 |  | 0.5 | 1 | 0.46 |  | 1 | 2 | 0.71 |  | 0.5 | 1 | 0.51 |  | 0.06 | 0.25 | 0.10 |
| *C. metapsilosis* | | | | | | | | | | | | | | | | | | | | | | | | | | | | | | | |
| CHIF-NET18 | 1 | 2 | 1.24 |  | 0.015 | 0.06 | 0.02 |  | 0.06 | 0.12 | 0.05 |  | 0.03 | 0.06 | 0.03 |  | 0.12 | 0.12 | 0.09 |  | 0.12 | 0.25 | 0.14 |  | 0.25 | 0.5 | 0.35 |  | 0.06 | 0.06 | 0.07 |
| CHIF-NET19 | 2 | 4 | 1.86 |  | 0.03 | 0.06 | 0.03 |  | 0.09 | 0.159 | 0.08 |  | 0.06 | 0.12 | 0.05 |  | 0.12 | 0.25 | 0.16 |  | 0.12 | 0.325 | 0.19 |  | 0.5 | 1 | 0.42 |  | 0.06 | 0.25 | 0.08 |
| CHIF-NET20 | 2 | 4 | 1.53 |  | 0.03 | 0.06 | 0.03 |  | 0.06 | 0.25 | 0.05 |  | 0.03 | 0.12 | 0.04 |  | 0.12 | 0.25 | 0.12 |  | 0.12 | 0.25 | 0.16 |  | 0.5 | 1 | 0.48 |  | 0.06 | 0.25 | 0.10 |
| CHIF-NET21 | 2 | 4.4 | 2.35 |  | 0.03 | 0.06 | 0.03 |  | 0.06 | 0.133 | 0.06 |  | 0.03 | 0.12 | 0.04 |  | 0.12 | 0.5 | 0.14 |  | 0.12 | 0.5 | 0.18 |  | 0.5 | 1 | 0.51 |  | 0.06 | 0.25 | 0.12 |
| CHIF-NET18-21 | 2 | 4 | 1.68 |  | 0.03 | 0.06 | 0.03 |  | 0.06 | 0.12 | 0.06 |  | 0.03 | 0.12 | 0.04 |  | 0.12 | 0.25 | 0.12 |  | 0.12 | 0.25 | 0.16 |  | 0.5 | 1 | 0.43 |  | 0.06 | 0.198 | 0.09 |
| *C. orthopsilosis* | | | | | | | | | | | | | | | | | | | | | | | | | | | | | | | |
| CHIF-NET18 | 1 | 32 | 2.81 |  | 0.03 | 2 | 0.10 |  | 0.12 | 0.45 | 0.14 |  | 0.12 | 0.25 | 0.11 |  | 0.5 | 1 | 0.39 |  | 0.5 | 1 | 0.68 |  | 0.5 | 1 | 0.55 |  | 0.06 | 0.12 | 0.08 |
| CHIF-NET19 | 2 | 48 | 4.12 |  | 0.06 | 2 | 0.15 |  | 0.25 | 0.5 | 0.18 |  | 0.12 | 0.5 | 0.15 |  | 0.5 | 1 | 0.52 |  | 0.5 | 1 | 0.55 |  | 0.5 | 1 | 0.52 |  | 0.06 | 2 | 0.13 |
| CHIF-NET20 | 16 | 38.4 | 6.45 |  | 0.25 | 1 | 0.16 |  | 0.12 | 0.25 | 0.10 |  | 0.12 | 0.25 | 0.11 |  | 0.5 | 0.5 | 0.41 |  | 0.5 | 0.6 | 0.40 |  | 0.5 | 0.5 | 0.43 |  | 0.06 | 0.06 | 0.06 |
| CHIF-NET21 | 8 | 32 | 5.10 |  | 0.25 | 1 | 0.14 |  | 0.12 | 0.25 | 0.12 |  | 0.12 | 0.25 | 0.10 |  | 0.5 | 0.5 | 0.34 |  | 0.5 | 0.5 | 0.39 |  | 0.5 | 1 | 0.46 |  | 0.06 | 0.12 | 0.07 |
| CHIF-NET18-21 | 2 | 32 | 4.13 |  | 0.06 | 2 | 0.13 |  | 0.12 | 0.25 | 0.14 |  | 0.12 | 0.25 | 0.12 |  | 0.5 | 1 | 0.41 |  | 0.5 | 1 | 0.51 |  | 0.5 | 1 | 0.50 |  | 0.06 | 0.12 | 0.08 |
| *C. parapsilosis* | | | | | | | | | | | | | | | | | | | | | | | | | | | | | | | |
| CHIF-NET18 | 0.5 | 1 | 0.68 |  | 0.015 | 0.03 | 0.02 |  | 0.06 | 0.12 | 0.06 |  | 0.06 | 0.12 | 0.05 |  | 0.5 | 1 | 0.55 |  | 1 | 2 | 1.08 |  | 0.5 | 1 | 0.54 |  | 0.06 | 0.25 | 0.11 |
| CHIF-NET19 | 0.5 | 2 | 0.87 |  | 0.015 | 0.06 | 0.02 |  | 0.12 | 0.12 | 0.08 |  | 0.06 | 0.12 | 0.06 |  | 1 | 1 | 0.77 |  | 1 | 2 | 1.14 |  | 0.5 | 1 | 0.62 |  | 0.12 | 0.25 | 0.13 |
| CHIF-NET20 | 0.5 | 2 | 0.70 |  | 0.008 | 0.03 | 0.01 |  | 0.03 | 0.12 | 0.04 |  | 0.03 | 0.12 | 0.03 |  | 0.5 | 1 | 0.53 |  | 1 | 2 | 0.79 |  | 0.5 | 1 | 0.47 |  | 0.06 | 0.25 | 0.10 |
| CHIF-NET21 | 0.5 | 4 | 0.77 |  | 0.008 | 0.06 | 0.02 |  | 0.06 | 0.12 | 0.05 |  | 0.03 | 0.12 | 0.04 |  | 0.5 | 1 | 0.59 |  | 1 | 2 | 0.87 |  | 0.5 | 1 | 0.50 |  | 0.06 | 0.12 | 0.10 |
| CHIF-NET18-21 | 0.5 | 2 | 0.74 |  | 0.015 | 0.06 | 0.02 |  | 0.06 | 0.12 | 0.06 |  | 0.06 | 0.12 | 0.04 |  | 0.5 | 1 | 0.59 |  | 1 | 2 | 0.96 |  | 0.5 | 1 | 0.53 |  | 0.06 | 0.25 | 0.11 |
| *C. tropicalis* | | | | | | | | | | | | | | | | | | | | | | | | | | | | | | | |
| CHIF-NET18 | 2 | 512 | 7.51 |  | 0.25 | 16 | 0.49 |  | 0.25 | 1 | 0.41 |  | 0.25 | 1 | 0.37 |  | 0.03 | 0.12 | 0.04 |  | 0.12 | 0.25 | 0.11 |  | 1 | 1 | 0.90 |  | 0.06 | 0.12 | 0.07 |
| CHIF-NET19 | 2 | 512 | 8.25 |  | 0.25 | 16 | 0.49 |  | 0.25 | 1 | 0.47 |  | 0.25 | 1 | 0.39 |  | 0.03 | 0.12 | 0.05 |  | 0.12 | 0.25 | 0.11 |  | 1 | 2 | 1.07 |  | 0.06 | 0.12 | 0.07 |
| CHIF-NET20 | 2 | 512 | 6.95 |  | 0.12 | 16 | 0.33 |  | 0.25 | 1 | 0.24 |  | 0.25 | 1 | 0.22 |  | 0.06 | 0.12 | 0.05 |  | 0.12 | 0.25 | 0.09 |  | 1 | 2 | 0.89 |  | 0.06 | 0.12 | 0.07 |
| CHIF-NET21 | 2 | 512 | 7.33 |  | 0.12 | 16 | 0.33 |  | 0.25 | 1 | 0.24 |  | 0.25 | 1 | 0.21 |  | 0.06 | 0.12 | 0.06 |  | 0.12 | 0.25 | 0.11 |  | 1 | 2 | 0.87 |  | 0.06 | 0.06 | 0.07 |
| CHIF-NET18-21 | 2 | 512 | 7.50 |  | 0.25 | 16 | 0.41 |  | 0.25 | 1 | 0.33 |  | 0.25 | 1 | 0.30 |  | 0.045 | 0.12 | 0.05 |  | 0.12 | 0.25 | 0.11 |  | 1 | 2 | 0.92 |  | 0.06 | 0.12 | 0.07 |
| *C. glabrata* | | | | | | | | | | | | | | | | | | | | | | | | | | | | | | | |
| CHIF-NET18 | 16 | 32 | 10.45 |  | 0.25 | 1 | 0.29 |  | 0.5 | 1 | 0.55 |  | 1 | 2 | 0.99 |  | 0.03 | 0.12 | 0.04 |  | 0.03 | 0.06 | 0.03 |  | 0.5 | 1 | 0.61 |  | 0.06 | 0.06 | 0.06 |
| CHIF-NET19 | 16 | 64 | 16.03 |  | 0.5 | 2 | 0.38 |  | 0.5 | 1 | 0.64 |  | 1 | 2 | 1.14 |  | 0.06 | 0.12 | 0.05 |  | 0.03 | 0.06 | 0.03 |  | 1 | 2 | 0.87 |  | 0.06 | 0.06 | 0.07 |
| CHIF-NET20 | 8 | 64 | 9.98 |  | 0.25 | 2 | 0.24 |  | 0.5 | 1 | 0.41 |  | 1 | 2 | 0.62 |  | 0.06 | 0.12 | 0.06 |  | 0.03 | 0.06 | 0.03 |  | 0.5 | 2 | 0.71 |  | 0.06 | 0.06 | 0.06 |
| CHIF-NET21 | 8 | 64 | 10.35 |  | 0.25 | 2 | 0.26 |  | 0.5 | 2 | 0.47 |  | 1 | 2 | 0.69 |  | 0.06 | 0.12 | 0.06 |  | 0.03 | 0.06 | 0.03 |  | 0.5 | 2 | 0.69 |  | 0.06 | 0.06 | 0.07 |
| CHIF-NET18-21 | 16 | 64 | 11.48 |  | 0.25 | 2 | 0.29 |  | 0.5 | 1 | 0.52 |  | 1 | 2 | 0.86 |  | 0.06 | 0.12 | 0.05 |  | 0.03 | 0.06 | 0.03 |  | 0.5 | 2 | 0.70 |  | 0.06 | 0.06 | 0.06 |
| *C. krusei* | | | | | | | | | | | | | | | | | | | | | | | | | | | | | | | |
| CHIF-NET18 | 64 | 64 | 51.83 |  | 0.5 | 0.5 | 0.35 |  | 0.25 | 0.5 | 0.28 |  | 0.25 | 0.5 | 0.31 |  | 0.25 | 0.25 | 0.18 |  | 0.06 | 0.12 | 0.05 |  | 1 | 1 | 0.75 |  | 16 | 16 | 9.97 |
| CHIF-NET19 | 64 | 128 | 48.61 |  | 0.5 | 1 | 0.44 |  | 0.5 | 0.5 | 0.37 |  | 0.5 | 1 | 0.43 |  | 0.25 | 0.5 | 0.27 |  | 0.06 | 0.12 | 0.06 |  | 1 | 2 | 1.19 |  | 16 | 32 | 13.56 |
| CHIF-NET20 | 32 | 64 | 39.01 |  | 0.25 | 0.65 | 0.32 |  | 0.185 | 0.5 | 0.18 |  | 0.25 | 0.5 | 0.29 |  | 0.12 | 0.25 | 0.16 |  | 0.03 | 0.06 | 0.03 |  | 1 | 2 | 0.80 |  | 8 | 16 | 9.05 |
| CHIF-NET21 | 32 | 64 | 38.94 |  | 0.25 | 0.5 | 0.29 |  | 0.25 | 0.5 | 0.17 |  | 0.25 | 0.5 | 0.25 |  | 0.12 | 0.25 | 0.16 |  | 0.03 | 0.06 | 0.04 |  | 0.5 | 1 | 0.59 |  | 8 | 16 | 8.42 |
| CHIF-NET18-21 | 64 | 64 | 45.62 |  | 0.5 | 0.5 | 0.35 |  | 0.25 | 0.5 | 0.25 |  | 0.25 | 0.5 | 0.32 |  | 0.25 | 0.5 | 0.19 |  | 0.06 | 0.12 | 0.05 |  | 1 | 2 | 0.82 |  | 16 | 16 | 10.34 |
